# Pain in the bipolar disorder: prevalence, characteristics and relationship with suicide risk*

**DOI:** 10.1590/1518-8345.4737.3463

**Published:** 2021-06-28

**Authors:** Ana Carolina Ferreira Rosa, Eliseth Ribeiro Leão

**Affiliations:** 1 Instituto Israelita de Ensino e Pesquisa Albert Einstein, Faculdade Israelita de Ciências da Saúde Albert Einstein, São Paulo, SP, Brazil.

**Keywords:** Pain, Pain Management, Bipolar Disorder, Suicide, Nursing, Mental Health, Dor, Manejo da Dor, Transtorno Bipolar, Suicídio, Enfermagem, Saúde Mental, Dolor, Manejo del Dolor, Trastorno Bipolar, Suicidio, Enfermería, Salud Mental

## Abstract

**Objective:**

to know the prevalence and characteristics of pain, to verify how pain management has been carried out by the health services, and to correlate suicide risk with pain intensity in patients with bipolar disorder.

**Method:**

an observational study with a quantitative approach. The study included people with bipolar disorder assessed by the McGill-Reduced Pain Questionnaire, Body Diagram, Visual Numerical Scale, and the Suicidal Ideation Scale (Beck).

**Results:**

the sample of 60 participants was mainly composed of women with a mean age of 40 years old and a mean psychiatric treatment time of approximately 13 years. Of these, 83% reported feeling pain at the time of the interview. Half of the participants indicated that pain interferes with routine and 80% did not receive care in health institutions. The main descriptors that qualify the painful experience were as follows: painful, heavy and sensitive for the sensory descriptors, tiring and punishing in the affective category. Suicide attempt was reported by 57% of the participants. There was a correlation between suicide risk and pain intensity.

**Conclusion:**

pain presented a high prevalence. Suicide risk was identified in more than half of the participants. Pain intensity showed a significant correlation with suicide risk.

## Abstract (pt)

**Objetivo:**

conhecer a prevalência e características da dor, verificar como o manejo da dor tem sido realizado pelos serviços de saúde e correlacionar o risco de suicídio com a intensidade da dor dos pacientes com transtorno bipolar.

**Método:**

estudo observacional com abordagem quantitativa. Participaram do estudo pessoas com transtorno bipolar avaliadas pelo Questionário de Dor McGill-reduzido, Diagrama Corporal, Escala Visual Numérica e a Escala de Ideação Suicida (Beck).

**Resultados:**

A amostra de 60 participantes foi composta principalmente por mulheres com idade média de 40 anos e tempo médio de tratamento psiquiátrico aproximado de 13 anos. Desses, 83% relataram sentir dor no momento da entrevista. Metade dos participantes indicou que a dor atrapalha a rotina e 80% não receberam cuidado nos equipamentos de saúde. Os principais descritores que qualificam a experiência dolorosa foram: dolorida, em peso e sensível para os descritores sensoriais, cansativo e castigante na categoria afetiva. A tentativa de suicídio foi relatada por 57% dos participantes. Foi observada correlação entre o risco de suicídio e a intensidade da dor. Conclusão: a dor apresentou prevalência elevada. O risco de suicídio foi identificado em mais da metade dos participantes. A intensidade da dor apresentou correlação significativa com o risco de suicídio.

## Abstract (es)

**Objetivo:**

conocer la prevalencia y las características del dolor, verificar cómo se ha realizado el manejo del dolor por parte de los servicios de salud y correlacionar el riesgo de suicidio con la intensidad del dolor en pacientes con trastorno bipolar.

**Método:**

se trata de un estudio observacional con abordaje cuantitativo. El estudio incluyó a personas con trastorno bipolar evaluadas mediante el Cuestionario de dolor de McGill – versión abreviada, el Diagrama Corporal, la Escala Visual Numérica y la Escala de Ideación Suicida (Beck).

**Resultados:**

la muestra de 60 participantes estuvo compuesta principalmente por mujeres con una edad promedio de 40 años y un tiempo promedio de tratamiento psiquiátrico de aproximadamente 13 años. El 83% de ellos manifestó sentir dolor en el momento de la entrevista. La mitad de los participantes indicó que el dolor interfiere en la rutina y el 80% no recibió atención en equipos de salud. Los principales descriptores que califican la experiencia dolorosa fueron: doloroso, pesadez y sensibilidad para los descriptores sensoriales, agotador y castigador en la categoría afectiva. El 57% de los participantes informó intento de suicidio. Se observó una correlación entre el riesgo de suicidio y la intensidad del dolor.

**Conclusión:**

el dolor tuvo una alta prevalencia. El riesgo de suicidio se identificó en más de la mitad de los participantes. La intensidad del dolor se correlacionó significativamente con el riesgo de suicidio.

## Introduction

The prevalence of pain in people with mental disorders has received little attention^(^ 1 ^)^. This fact surprises us, since people with Bipolar Disorder (BD) are at significant risk for a series of painful physical diseases^(^ 2 ^)^.

BD is a cyclic and heterogeneous disease in its clinical presentations. According to the new International Classification of Diseases (ICD-11, 2018), it is characterized by the presence of mania/hypomania, depression and mixed episodes^(^ 3 ^)^. BD prevalence in the classic forms is around 2.4% and the impact on people’s lives is quite significant^(^ 4 ^)^.

In general, the onset of the disease manifests in youth, it takes a mean of eight years for people to be diagnosed and treated properly, with unipolar depression being the most frequent diagnostic error, which generates great losses for the individuals, their family and society^(^ 3 ^)^.

The first choice treatment is with mood stabilizing drugs, anticonvulsants and atypical antipsychotics. The combination of drugs with psychosocial interventions such as psychotherapy (individual or group) and psychoeducational groups has been shown effective^(^ 5 ^)^.

In addition, the risk of mortality in people with BD is twice that of the general population^(^ 6 ^)^. It is estimated that 50% of the individuals with BD attempt suicide at least once in their lives, and nearly 11% to 19% reach their goal^(^ 7 ^)^. It is also known that the risks for suicidal behavior and functional losses are greater in people not treated in the different care dimensions (biopsychosocial)^(^ 8 ^)^.

When compared to those without severe mental disorders, people with BD are more likely to experience conditions that cause pain and, concomitantly, receive less adequate care to control it^(^ 2 ^)^. This data is relevant since pain is also associated with the worsening of the psychiatric symptoms^(^ 1 ^)^. Mental health experts report barriers that limit their ability to treat physical comorbidity^(^ 9 ^)^.

Listening and valuing the pain complaint from people with mental disorder is important; however, it is common to interpret pain complaints as always related to emotional conflicts, instead of interpreting the painful symptom as an expression of a possible correlation of psychological events with organic events^(^ 9 ^)^.

Scientific development has advanced and resulted in the creation of several disciplines, which has brought positive and negative aspects to health care. If, on the one hand, compartmentalizing knowledge in disciplines allowed men to achieve extremely specific knowledge on certain themes, on the other hand it generated not only the division of labor, but over-specialization, which leads to the loss of the generalist view and to fragmentation of knowledge^(^ 10 ^-^ 11 ^)^.

In this way, we observe professionals specialized in physical, social, mental and spiritual complaints that, however, lack an integrating view of the human being that guides assistance in an adequate way in meeting human needs.

Systematic pain assessment must be performed as part of the management of mental disorders, and pain should be monitored during the course of the treatment^(^ 12 ^)^. Therefore, it is essential that the treatment of the multidisciplinary team in mental health equipment seeks to provide adequate assessment and treatment of pain in people with BD. The literature is scarce and, above all, it lacks recent articles on this topic.

Thus, this study aimed to: 1) Know the prevalence and characteristics of pain in patients with BD; 2) Verify how pain management has been carried out by the health services from the perspective of patients with TB; and 3) Correlate suicide risk with pain intensity in patients with BD.

## Method

This is an observational and descriptive study, with a quantitative approach, developed in the Psychosocial Care Center (*Centro de Atenção Psicossocial*, CAPS) for Adults III in Paraisópolis, South district of the city of São Paulo, SP, Brazil, where one of the researchers worked. The Psychosocial Care Centers (CAPS) are strategic services aiming at propitiating access and providing comprehensive treatment to mental health in a regionalized manner. Data collection lasted for three months (March to May 2019).

Within the population with a BD diagnosis being monitored in the service (63), only three patients did not agree to participate. The inclusion criteria to be followed were having a BD diagnosis, being followed up in the CAPS for Adults III in Paraisópolis, and being 18 years old or older. And no participant met the clinically-assessed exclusion criterion, which was having psychomotor agitation with risk of self-aggression and/or heteroaggression at the time of the interview. Thus, 60 individuals composed the sample for convenience at the end of the study.

The research project was approved by the Research Ethics Committee (*Comitê de Ética em Pesquisa*, CEP) of the Albert Einstein Israeli Hospital - SP, approval date: January 9^th^, 2019, CAAE: 04083218.0.0000.0071, opinion No.: 3,109,674; and was also approved by the CEP of the Municipal Health Secretariat of São Paulo, approval date: January 30^th^, 2019, CAAE: 04083218.0.3001.0086, opinion No.: 3,125,885, according to resolution 466/12 of the National Health Council^(^ 13 ^)^. All the study participants agreed to sign the Free and Informed Consent Form to participate in the research, getting a copy of equal content and tenor signed by the researcher.

Instruments were used to investigate sociodemographic and clinical data, characterization of pain and suicide risk for patients with Bipolar Disorder.

The population characterization form consisted of identification, religion and marital status data, time of knowledge and treatment of mental disorders and the existence of other diseases.

Pain assessment consisted in the pain assessment form, composed of: Body Diagram, multiple choice questions regarding frequency, duration, factors of pain improvement and worsening, care of the health network, impact on the routine, McGill Pain Questionnaire (MPQ) – reduced version and the Visual Numerical Scale (VNS).

The body diagram for pain localization consists of the schematic representation of the human body, front and back, on which the patient indicates where pain is located. The pain frequency questions range from feeling pain every day to feeling it once a month.

Pain duration has been assessed considering that painful sensation is “direct/without stopping” or if it “stops and then returns”, and the time (minutes, hours, days or constant). The last questions for this stage of the questionnaire are in relation to what causes pain to improve or worsen, if pain hinders the routine and, finally, if the interviewee received support in the health network in relation to pain.

The McGill Pain Questionnaire (MPQ) – reduced version aims to provide qualitative pain measures that can be statistically analyzed^(^ 14 ^)^. The MPQ was designed to evaluate the multidimensional nature of the pain experience and proved to be a reliable, valid and consistent measurement tool^(^ 15 ^)^. In this research, the MPQ translated and adapted to the Portuguese^(^ 16 ^)^ was used, with its original version being elaborated in 1975^(^ 14 ^)^.

The multidimensional instrument evaluates several aspects of pain by means of 15 words (descriptors) that the patients choose to express their pain. The descriptors are divided into two groups: sensory and affective. The numerical index of descriptors is the number of words chosen by the patients to characterize their pain, each of these words receives a value between 0 and 3 points, where 0 corresponds to “no pain” and 3 corresponds to “severe” pain. The calculated scores are as follows: sensory score (it varies from 0 to 33), affective score (it ranges from 0 to 12), and total score (it varies from 0 to 45)^(^ 16 ^)^.

The Visual Numerical Scale (VNS) is a one-dimensional instrument for assessing pain intensity. The VNS consists of a ruler divided into eleven equal parts, numbered successively from 0 to 10. The interviewees referred to the equivalence between the intensity of their pain and a numerical classification, being painless (0), mild (1 to 3), moderate (4 to 7), and intense/excessive pain (8 to 10)^(^ 17 ^)^.

The third instrument used was the Beck Suicidal Ideation (BSI) Scale, which assesses the presence of suicidal ideation, as well as the severity of suicide ideas, plans and desires. There is no specific cut-off point, nor levels, because a score above 0 already indicates the existence of suicidal ideation and deserves attention^(^ 18 ^)^.

The BSI consists of 21 items and the first 19 items, presented with three alternative answers, reflect gradations of the severity of suicidal-related desires, attitudes and plans. The last two items, not included in the final informative score, provide important support on the patient regarding the number of previous suicide attempts and the seriousness of the intention to die in the last^(^ 18 ^)^.

In this research we used the translated and adapted version for Portuguese^(^ 18 ^)^; its original version is the *Scale for Suicide Ideation*, SSI, presented in 1979^(^ 19 ^)^. The quality of the psychometric characteristics of the SSI was investigated, being the instrument used in research and clinical work, based on adult psychiatric patients, inpatients and outpatients^(^ 20 ^)^.

According to the criteria of the original manual, the administration and score of the BSI can be under the responsibility of a paraprofessional, while the interpretation must be borne by the professional with clinical experience. In this study, the authors themselves were responsible for data collection and analysis of statistical data, since they contemplate the prerequisites of the manual. We emphasize that the interpretation of the results of this instrument was performed through the description of the statistical results, and not the subjective interpretation of each domain of the scale^(^ 18 ^)^.

The researchers programmed a schedule for all BD patients under follow-up in the CAPS for Adults III in Paraisópolis. The potential participants were already under follow-up in the CAPS; thus, to optimize data collection, and have no cost to them, we prioritized scheduling for data collection on the days that coincided with treatment in the CAPS.

At the scheduled time, the potential participants were referred to the office of the CAPS for Adults III in Paraisópolis. After evaluation of the inclusion and exclusion criteria, the objectives of the study were explained.

After reading and signing the Free and Informed Consent Form (FICF), each participant received a copy of the three research instruments to accompany the reading during the interview. The researcher read the instrument, inflaming the answers given by the interviewee. The interviewer reread the question not understood, paused, without any other explanation or use of a synonym. At the end of the interview, the participant’s doubts were clarified.

The quantitative variables were described through mean, standard deviation, median, interquartile interval and extreme values. The qualitative variables were described by means of absolute frequency and percentage^(^ 21 ^)^.

The distribution of the suicidal ideation data, measured by the total Beck Suicidal Ideation scale, was quite asymmetric, with a concentration of zero values in the sample. The values observed varied between zero and 27, with a median of zero, first quartile zero, and third quartile 11. With this distribution, it was necessary for us to be concerned not only with a model that included an asymmetric distribution but also with one that included the excess of zeros in the sample. Because it is a discrete score, that is, without decimal places, a candidate distribution was the negative binomial distribution, suitable for asymmetric behavior and discrete data.

To contemplate zero inflation we use a mix model, with the aid of the Generalized Additive Models for Location, Scale and Shape (GAMLSS) from the R package, which considers a negative binomial distribution adjusted for excess zeros^(^ 22 ^-^ 23 ^)^. The verification of the quality of fit was done by means of the graphic analysis of the residuals of the model, which showed satisfactory behavior in order to be able to draw inferential conclusions from the adjusted model.

The results were presented by estimated means, 95% confidence intervals in the groups and p-values for the comparisons. The analyses were performed with the aid of the Statistical Package for the Social Sciences (SPSS)^(^ 24 ^)^, R^(^ 23 ^)^ and GAMLSS^(^ 22 ^)^ packages, and considering a 5% significance level.

## Results

The sample consisted of 60 individuals with Bipolar Disorder being followed up in the CAPS for Adults III in Paraisópolis. As shown in Table 1, the mean age of the participants was 40 years old. The majority of the sample comprised women.

**Table 1:** Sociodemographic and clinical characteristics of the BD* patients. São Paulo, SP, Brazil, 2019

Variables	Total
Age (years old)	
Mean (SD^†^)	40.92 (11.36)
Median [IQR^‡^]	40.00 [32.00; 51.25]
Minimum - Maximum (n)	21.00 -65.00 (60)
**Gender - n (%) (N=60)**	
Female	43 (71.7)
Male	17 (28.3)
How long have you known you have BD* (years)	
Mean (SD^†^)	9.20 (7.82)
Median [IQR^‡^]	7.50 [2.00; 15.00]
Minimum - Maximum (n)	0.08 -30.00 (60)
**How long have you been on psychiatric treatment (years)**	
Mean (SD^†^)	13.36 (9.36)
Median [IQR^‡^]	12.00 [7.00; 20.00]
Minimum - Maximum (n)	0.08 -39.00 (60)
**Number of psychiatric hospitalizations**	
Mean (SD^†^)	7.90 (9.94)
Median [IQR^‡^]	4.00 [1.75; 10.00]
Minimum - Maximum (n)	0.00 -40.00 (60)
**Do you have another disease or health problem - n (%) (N=60)**	
No	37 (61.7)
Yes	13 (21.7)
Don't know	10 (16.7)

* BD = Bipolar Disorder;

† SD = Standard Deviation;

‡ IQR = Interquartile Range

The mean psychiatric treatment time was approximately 13 years, but the mean time of knowledge on the BD diagnosis was 9 years. Throughout their lives, the participants experienced 8 psychiatric hospitalizations (mean).

Table 2 shows the prevalence and characterization of pain. Of the sixty study participants, fifty reported feeling pain at the time of the interview (83%), with 60% of the participants feeling musculoskeletal pain. The spinal cord was the most reported, and 44% of the participants experience pain every day.

**Table 2:** Prevalence and characterization of pain in the BD* patient. São Paulo, SP, Brazil, 2019

Variables	Total
**Presence of pain - n (%) (N=60)**	
No	10 (16.7)
Yes	50 (83.3)
**Pain intensity (0 to 10) (N=60)**	
Mean (SD^†^)	5.68 (3.31)
Median [IQR^‡^]	6.00 [3.75; 8.00]
Minimum - Maximum	0.00- 10.00
**Main pain location - n (%) (N=50)**	
Cephalic	8 (16.0)
Thoracic	4 (8.0)
Spinal cord	21 (42.0)
Abdomen	2 (4.0)
UULL^§^	7 (14.0)
LLLL^||^	8 (16.0)
**Pain frequency- n (%) (N=50)**	
Every day	22 (44.0)
Three times a week	5 (10.0)
Twice a week	6 (12.0)
Once a week	7 (14.0)
Once or twice a month	10 (20.0)
**What do you do to alleviate your pain? - n (%) (N=50)**	
Nothing	7 (14.0)
I take a medication that I buy directly in the pharmacy	4 (8.0)
I take a medication prescribed by the doctor	12 (24.0)
I seek some health service	4 (8.0)
I use medicinal herbs	1 (2.0)
I turn to religion	2 (4.0)
I rest	6 (12.0)
I walk	2 (4.0)
I talk with someone	5 (10.0)
Others	7 (14.0)
**What makes your pain worse? - n (%) (N=50)**	
Resting	8 (16.0)
Walking	18 (36.0)
Getting nervous	16 (32.0)

* BD = Bipolar Disorder;

† SD = Standard Deviation;

‡ IQR = Interquartile Range;

§ UULL = Upper Limbs;

|| LLLL = Lower Limbs

In the pain intensity measured by the Visual Numerical Scale (VNS), the values varied from zero to ten, with a median of six (moderate pain).

Table 3 shows how pain management has been carried out from the perspective of the patient with BD. An expressive portion of the participants did not receive pain care and did not feel welcomed in the health services. Half of the participants indicated that pain disturbs their routine.

**Table 3:** Handling of pain in the patient with BD* in the health services. São Paulo, SP, Brazil, 2019

Variables	Total
**Did you receive pain care in any health service? - n (%) (N=50)**	
Yes, in the Psychosocial Care Center	1 (2.0)
Yes, in the Basic Health Unit	4 (8.0)
Yes, in Ambulatory Medical Assistance	1 (2.0)
Yes, in the Emergency Service	2 (4.0)
No	40 (80.0)
Others	2 (4.0)
**What other care did you receive - n (%) (N=2)**	
Orthopedist - Outpatient clinic	1 (50.0)
Private orthopedist	1 (50.0)
**Reception at the time of the pain complaint - n (%) (N=50)**	
No	42 (84.0)
Yes	8 (16.0)
**Pain disrupts your routine - n (%) (N=50)**	
No	25 (50.0)
Yes	25 (50.0)

* BD = Bipolar Disorder

Table 4 shows the answers of the subjective pain experience using the McGill pain questionnaire - reduced version. The median value of the sensory score was 12.5; of the affective score 6.0; and finally, the median value of the total score was 18.0.

**Table 4:** Pain assessment by the McGill pain Questionnaire. São Paulo, SP, Brazil 2019

McGill	Intensity							
	0*		1^†^		2^‡^		3^§^	
	N	%	N	%	N	%	N	%
**Sensory**								
Throbbing	21	35.0%	15	25.0%	14	23.3%	10	16.7%
Stabbing	24	40.0%	9	15.0%	21	35.0%	6	10.0%
Acute	29	48.3%	8	13.3%	11	18.3%	12	20.0%
Colic	54	90.0%	0	0.0%	4	6.7%	2	3.3%
Bite	46	76.7%	6	10.0%	6	10.0%	2	3.3%
Heat-burning	33	55.0%	7	11.7%	10	16.7%	10	16.7%
Painful	13	21.7%	6	10.0%	21	35.0%	20	33.3%
Heavy	17	28.3%	4	6.7%	18	30.0%	21	35.0%
Sensitive	20	33.3%	5	8.3%	16	26.7%	19	31.7%
Breaking	29	48.3%	14	23.3%	11	18.3%	6	10.0%
**Affective**								
Tiring-exhausting	16	26.7%	7	11.7%	14	23.3%	23	38.3%
Sick	46	76.7%	2	3.3%	7	11.7%	5	8.3%
Frightened	29	48.3%	6	10.0%	10	16.7%	15	25.0%
Punishing-tormenting	14	23.3%	5	8.3%	19	31.7%	22	36.7%

* 0 = No pain;

† 1 = Light pain;

‡ 2 = Mild pain;

§ 3 = Severe pain

It is observed that the most scored descriptors that qualify the painful experience were the following: painful, heavy and sensitive for sensory descriptors, tiring/exhausting and punishing/tormenting in the affective category.

As for the answers to the Beck suicidal ideation scale questionnaire, the total score (suicidal ideation and planning) varied from 0 to 28. Approximately 57% of the respondents attempted suicide at least once with moderate to high desire.

In Table 5, we observe the statistically significant relationship between pain measured by the Visual Numerical Scale (VNS) and suicidal ideation.

**Table 5:** Relationship between pain and suicidal ideation. São Paulo, SP, Brazil, 2019

	With pain (n=50)		Total (n=60)	
	Means ratio (95 CI*)	p-value^†^	Means ratio (95 CI*)	p-value^†^
Pain by the Visual Numeric Scale - VNS^‡^	1.127 (1.006;1.263)	0.044	1.145 (1.003;1.307)	0.049
Pain by McGill (MPQ^§^) - Sensory score	1.039 (0.996;1.083)	0.085	1.040 (0.996;1.087)	0.084
Pain by McGill (MPQ^§^) - Affective score	1.005 (0.919;1.100)	0.907	1.145 (0.920;1.102)	0.883
Pain by McGill (MPQ^§^) - Total Score	1.025 (0.990;1.060)	0.167	1.026 (0.990;1.064)	0.163

* CI = Confidence Interval;

† p-value = Significance value;

‡ VNS = Visual Numeric Scale;

§ MPQ = McGill Pain Questionnaire

The data in Table 5 further demonstrate that, with each addition of a point on the pain scale, a mean increase of 14.5% in the mean suicidal ideation score is expected (95% CI: 0.3% to 30.7%, p-value=0.049) for the total sample. For the sample with pain, this relationship is also significant: with each one-point increase on the pain scale, we expect a 12.7% increase in the mean suicidal ideation score (95% CI: 0.6% to 26.3%, p-value=0.044), that is, the greater the pain, the higher the suicidal ideation score.

## Discussion

In this study on pain, the sample consisted of 60 people with Bipolar Disorder being followed up at the CAPS for Adults III in Paraisópolis.

The first observation to be made is the predominance of women in the sample, different from studies of BD prevalence, which found no significant difference in the distribution by gender^(^ 25 ^-^ 26 ^)^. One possible explanation is the greater female demand for the health services, which is in line with other studies^(^ 27 ^-^ 28 ^)^, since being female was a predictor of greater demand for health care^(^ 28 ^)^.

Our results corroborate with the studies that identified the delay and challenges in the diagnosis of Bipolar Disorder^(^ 3 ^)^. It is known that 69% of patients are not diagnosed correctly, and end up consulting a mean of four physicians before receiving the proper diagnosis^(^ 5 ^)^.

Late diagnosis and the numerous hospitalizations experienced by the interviewees alert us on the risk for increased morbidity and mortality of these individuals, since the consequences of the delay in diagnosis are associated with a lower probability of adequate drug treatment and with increased rates of suicide and hospitalization^(^ 5 ^)^, which ends up reflecting on the prognosis and quality of life of these people.

Studies in the city of São Paulo indicate a prevalence of pain of 28.1% in household telephone surveys, rising to 43%, when data were obtained through face-to-face interviews^(^ 29 ^-^ 30 ^)^. One of the few studies on the prevalence of pain in people with Bipolar Disorder found that one out of four individuals with BD is affected by chronic pain, and migraine was three times more common than in the general population^(^ 12 ^)^.

The prevalence of pain in homeless people is higher (82.6%) and more similar to the findings of this study, showing that neglected populations tend to have more critical conditions when researched^(^ 31 ^)^.

In this study, nearly all of the participants reported pain at the time of the interview, the majority being musculoskeletal in nature and with daily frequency. They indicated improvement in pain when taking the medication prescribed by the physician and worsening when walking and when they get nervous. Half of the respondents indicated that pain disrupts their routine.

In relation to our results, it is known that musculoskeletal pain is prevalent in the population, worsening with increased load (walking, lifting, lowering, carrying objects) and improving with rest and with the use of medications^(^ 32 ^-^ 33 ^)^. In this study, worsening of pain is also associated with feeling nervous, a result that was also identified in the study of schizophrenic people with chronic pain^(^ 33 ^)^.

The duration and frequency of pain influence people’s lives. Frequent pain wears down those who feel it, demands the search for health services, financially burdens the family, consumes energy for activities of daily living and, possibly, the disposition for psychosocial rehabilitation activities^(^ 33 ^)^.

In this sample, the mean pain intensity was presented as moderate, while the most scored descriptors that qualify the interviewees’ painful experience were the following: painful, heavy and sensitive for sensory descriptors, tiring/exhausting and punishing/tormenting in the affective category.

More severe pain tends to be more disabling, and the intensity of the complaint is an important factor in reporting to professionals and family members, in the conduction of the therapy, even in the decision to refer the patient to other specialties, or not^(^ 34 ^)^.

Pain-related sensory quality often indicates the pathogenesis of the condition. Tight, colic, torsional pain often indicates processes in hollow viscera; burning, heat and shock pain is often related to conditions where there are neuropathies; pain described as painful and heavy often refers to situations involving deep muscular structures^(^ 13 ^-^ 15 ^)^. Indications that are related to our results from sensory descriptors, since musculoskeletal pain was the most frequent in the sample. The affective qualities of pain indicate the emotional component that every painful condition has^(^ 16 ^,^ 33 ^)^.

The findings that characterized pain in our sample collaborated to elucidate how the painful experience involves the sensitive, emotional, cognitive and sociocultural aspects, and consequently invite us to rethink the importance of assessing pain in people with BD^(^ 34 ^)^.

An expressive portion of the participants did not feel welcomed in the health service when presenting the pain complaint, a result that reinforces the non-prioritization of pain assessment of people with mental disorders^(^ 34 ^)^, in addition to alerting us to the challenges faced by health professionals in the care of people with BD in relation to pain complaints.

During the interview, many participants were surprised by the questions because they were not used to this type of care, a reaction that drew our attention since, in some cases, the verbalization of pain provides relief from the painful sensation^(^ 34 ^)^.

From these findings, it is possible to emphasize the relevance of the patients communicating their painful experience to the health professionals for understanding the condition. The challenge of the professional in identifying the complaint can be related to the way the investigation of symptoms is carried out.

In addition to this possibility, we cannot ignore the devaluation of the pain complaint in the stigma surrounding people with Bipolar Disorder. People’s behavior starts to be seen as unpredictable, fickle and problematic, arouses anguish, fear and anxiety, because it is believed to be facing something unknown, which is out of control, putting professionals in the place of impotence and generating frustration^(^ 35 ^)^.

The care of the mental health user, in general, usually occurs in the most advanced stages of the disease, with greater difficulty in treatment and, consequently, less investment, which implies the deterioration of the health conditions and reduced quality of life^(^ 36 ^)^.

Regarding suicide risk, more than half of the participants attempted suicide at least once in their lives, a finding that corroborates the significant suicide risk of people with BD^(^ 8 ^)^.

Another aspect related to suicidal behavior in bipolar patients has been demonstrated in studies which reveal that suicide risk in this population can be higher during the early years of the disease. Thus, a delay in diagnosis and, consequently, in mood stabilization, could increase suicide risk^(^ 4 ^,^ 8 ^)^.

BD is a serious mental disorder associated with suicidal behavior. Hence, understanding the neurobiological and clinical correlates of suicidal behavior can contribute to a reduction of the suicide rates in this population.

The correlation between pain intensity and suicide risk was statistically significant: while pain intensified, the risk of suicide in the sample increased. We have not identified studies in the literature with this relationship in people diagnosed with BD, a scenario that reinforces the need for studies of pain in the field of mental health.

Among the limitations of the study, we have the small sample of a single service and the scarcity of information on the characteristics of the disease, such as the possibility of the pain report varying according to the phases and polarities of Bipolar Disorder, which deserves to be investigated in the future. The number of participants is also related to one of the main difficulties for treating people with BD, the fact that approximately 50% interrupt treatment at least once, while 30% of them do so at least twice^(^ 3 ^,^ 14 ^)^. Due to their magnitude, non-adherence or low adherence to therapy are public health problems^(^ 3 ^)^.

Although the theme of pain in people with mental disorders is relevant and present, it is still little explored in research in our country. Therefore, this study displays relevant contributions to the study of pain in vulnerable and, above all, neglected populations. This is a pioneering study that expands the discussion for the mental health professionals who serve this population and who sometimes do not include pain assessment in their daily work. The findings can also support rich discussions in teaching and point to the expansion and necessary further studies on pain in this and other mental disorders.

Pain management in the mental health field can contribute to deeper and more sophisticated insights on pain syndromes, and for psychiatric morbidity in general, regardless of people’s state of pain, in order to thus promote powerful interactions between different specialties^(^ 1 ^)^.

## Conclusion

The study revealed moderate pain of high prevalence in patients with BD, mainly musculoskeletal, which improves with pharmacological treatment, whose worsening is related to physical activity and emotional aspects. A significant portion of the participants reported having complained of pain and not having received care in the health services. Suicide risk was identified in more than half of the participants and showed a significant correlation with pain intensity.
